# Poisson statistics-mediated particle/cell counting in microwell arrays

**DOI:** 10.1038/s41598-018-20913-0

**Published:** 2018-02-05

**Authors:** Christian D. Ahrberg, Jong Min Lee, Bong Geun Chung

**Affiliations:** Department of Mechanical Engineering, Sogang University, Seoul, Republic of Korea

## Abstract

Precise determination of particle or cell numbers is of importance for a wide array of applications in environmental studies, medical and biological applications, or manufacturing and monitoring applications in industrial production processes. A number of techniques ranging from manual counting to sophisticated equipment (e.g., flow cytometry) are available for this task. However, these methods are either labour intensive, prone to error, or require expensive equipment. Here, we present a fast, simple method for determining the number density of cells or microparticles using a microwell array. We analyze the light transmission of the microwells and categorize the microwells into two groups. As particles/cells contained in a microwell locally reduce the light transmission, these wells displayed a lower average transmission compared to unoccupied microwells. The number density of particles/cells can be calculated by Poisson statistics from the ratio of occupied to unoccupied microwells. Following this approach, the number densities of two different types of microparticles, as well as *HeLa* and *E*. *Coli* cells, ranging over four orders of magnitude were determined. Through the microwell array defined by microfabrication, a simple image recognition algorithm can be used with the formation of aggregates or irregular shaped samples providing no additional difficulty to the microwell recognition. Additionally, this method can be carried out using only simple equipment and data analysis automated by a computer program.

## Introduction

Precise determination of particle and cell numbers is of importance for a variety of industrial, clinical, and environmental applications. For example, increasing air pollution and fine particulate matter concentrations are a problem in many cities around the world^1,2^. In biological applications, the number of red blood cells^3,4^, bacteria^5^, neurons^6^, or uptake of nanoparticles^7^ has to be determined. Knowing the number of particles in the engine oil is of importance in predictive maintenance of aircraft jet engines^8^, or in monitoring clean rooms for microfabrication^9^. For all of those examples, a quick, simple, and reliable counting method is desirable for point-of-care applications^10^.

Conventionally bacteria are counted by smearing a sample onto a culture-substrate and counting the number of colonies after culturing^11,12^. However, this method has a number of disadvantages. Firstly, counting bacteria using this method is labor intensive and can take several days due to the necessary time for culture. Secondly, the method is not able to count dead bacteria. Lastly, competitive growth makes counting of slow growing species difficult. Alternatively bacteria, particle or eukaryotic cell samples can be manually counted using a microscope^13^. Although automated image processing techniques have replaced manual counting^14–16^, the method is still prone to error. Especially irregular-shaped samples or large aggregates are difficult to count accurately using optical methods. These advantages can be countered by dividing the sample using an array of microwells, cells can be counted by first recognizing the microwells by image recognition, and then the individual cells within the wells^17^. While this method is fast and reliable, it requires images with significant magnification and resolution to identify both microwells and cells. In 1953, Wallace H Coulter developed the first counting method that did not rely on optical detection^18^. An aperture is placed between two electrodes, creating a sensing area through the aperture. If a particle passes through the sensing area, a short-term change in impedance across the aperture is created. Through the simple setup and facile automation of measurements, the method quickly gained popularity. Resistive pulse sensors based on the working principle of Coulter Chambers were developed to detect nanometer scaled objects^19^. Despite the simplicity and label free detection, resistive pulse sensors are limited through their aperture regarding sensitivity as well as experimental throughput. To improve the signal to noise ratio and sensitivity of resistive pulse sensors, Wu *et al*. coupled them with flow cytometry^20^. In flow cytometry, cells or particles are focused by two neighboring sheath flows inside a microfluidic channel. The focused particles and cells can then be counted through a laser focused on the middle of the channel^21^. Detection is either achieved by measuring the fraction of light that is blocked from passing the detection volume by the particles^22^, or alternatively the light scattered by the particles can be measured^23^. Although flow cytometry has proven to be a fast and accurate counting method for a variety of samples, the main downside of flow cytometry lies in the sensitive alignment of the sample, the light source, and detector. This increases system complexity and costs.

In the quantification of DNA, a limiting dilution polymerase chain reaction (PCR), also known as digital PCR (dPCR), is becoming widely applied due to its accurate determination of initial DNA concentrations^24,25^. The method is based on dividing a sample into several subsamples in such a manner that each subsample contains less than one molecule of DNA on average. The sample is analyzed by PCR and the subsamples showing amplification are determined. Finally, the initial DNA concentration can be determined with great accuracy using Poisson statistics. This method can further be used to count DNA labelled nanoparticles^26^. In this paper we describe a novel method for counting micrometre sized particles or cells using a microwell array. Similar to dPCR, the sample is divided into several sub samples by the microwell array, microscope images of the well array are taken and the transmission of each well determined. As wells containing particles or cells have a lower light transmission, wells can be categorized into two groups according to light transmission: wells containing at least one particle/cell, and unoccupied wells. Now the number of particles/cells present in the sample can be determined using Poisson statistics. We demonstrate our method using two different types of microparticles. Furthermore, *E*. *Coli* and *HeLa* cell counting is shown in this paper. To facilitate easy differentiation between occupied and unoccupied wells, the cells are stained fluorescent, or stained using Trypan blue. This method allows a simple, inexpensive, and fast determination of cell and particle counts over several orders of magnitude. The image analysis can easily be automated by computer programs. As the design and dimensions of the microwells are defined by microfabrication, they can be detected by a simple image detection, such as Hough transform. While conventional methods have to account for irregular shaped samples and aggregates to be recognized, our method does not require this complication as only the microwells have to be recognized. For counting the average, light transmission of microwells is determined and number densities are determined by Poisson statistics. In our method, neither the detection of microwells nor the classification of wells is influenced through the formation of aggregates or irregular shaped samples.

## Material and Methods

### Device fabrication

For fabrication of the poly(dimethylsiloxane) (PDMS) microwell array chips, a design was created using Autocad (Autocad 2017, Autodesk, USA) and transferred to transparent film mask. Silicon masters were created in a standard soft lithography process^27^. Briefly, SU8-50 (Microchem Corp., USA) was spin-coated onto silicon wafers (Wangxiang Silicon-Peak Electronics, China) in a thickness of 25 µm. After ultra-violet (UV) illumination through the previously made film mask, the silicon masters were developed using SU-8 developer (Microchem Corp., USA). The detailed fabrication process of the silicon master can be found in Supplemental Table S1. PDMS (10:1 Monomer: curing Agent, Microchem Corp., USA) was poured onto the silicon masters and cured in an oven at 85 °C for one hour. The PDMS was carefully peeled from the silicon master and individual devices separated by cutting with a surgical blade.

### Sample loading

The microwell arrays were loaded by first treating the PDMS devices in oxygen plasma (1 V Generator voltage, Cute Series, FemtoScience, Korea) for one minute to decrease the hydrophobicity of the PDMS. This leads to the formation of hydrophilic silanol groups on the surface of the PDMS^28^. Next 10 µL of sample solution were carefully pipetted onto the area of the microwell array. To remove air trapped in the wells, the device was placed in a vacuum chamber at 0.4 MPa for one minute. Afterwards, the PDMS well arrays were covered using a microscope slide (Marienfeld, Germany) and excess solution was carefully removed from the edge of the PDMS device using a paper towel.

Dilution series containing fluorescent labeled polystyrene microparticles (Diameter 10.2 µm, Spherotech, USA) or silica coated magnetic microparticles (diameter 1–5 µm, Bioneer, Korea) in water were used. Further a culture of green fluorescent protein (GFP) transfected *HeLa* cells was directly used as a sample without further sample preparation. Lastly, an *E*. *Coli* culture was used as a sample. For labelling, 250 µL of the *E*. *Coli* culture solution was diluted with 250 µL of water. Afterwards, 500 µL of 0.4% Trypan blue solution (Invitrogen, Germany) or 1 µL of 100 µM BacLight Green Bacterial Stain (Molecular Probes, USA) in DMSO was added and the sample incubated for 15 minutes at room temperature before loading. The loaded microwell arrays were imaged under a fluorescent microscope (IX73, Olympus, Japan). The illumination of light intensity and shutter time of the camera were manually adjusted for each device to provide a clear differentiation between occupied and unoccupied microwells. From each of the 36 panels of the microwell array one image was taken for analysis with a custom written Python program (Information on code in Supplemental Materials). In addition, microparticle and cell samples were measured using a commercial cell counter (Eve Automatic cell counter, NanoEnTek, Korea) for comparison. For counting by the cell counter, cells were stained using 0.4% Trypan blue solution according to manufacturer’s instructions.

### Image analysis and counting algorithm

The images taken by the microscope were cropped to the area of the panel containing wells. The images were analyzed using a custom written Python program. The program first removed noise by taking the average value of pixels in a kernel and assigning the average value to the middle pixel. Next, the Hough transform was used for finding the location of the wells, for this the open openCV, a open source package, was used (OpenCV team, www.opencv.org). Once wells were identified, a well array was fitted by extracting the bottom row and most left row from the wells found by the Hough transform and afterwards fitting the known 30 by 30 well array using these coordinates. Images were converted to grayscale and the average pixel intensity value found for each of the fitted wells. Finally, a histogram of average well intensity was plotted, a threshold for classifying occupied and unoccupied wells defined, and lastly the average number of cells/microparticles calculated using Poisson statistics.

## Results and Discussion

### Filling of microwell array

**Figure 1:**
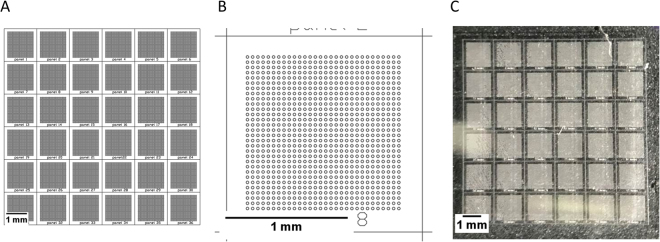
Design of the PDMS microwell array chip. CAD drawing of the entire microwell array chip, containing 36 individual panels (**A**). Drawing of an individual panel, containing 900 microwells in a 30 by 30 square arrangement (**B**), and photograph of the produced PDMS microwell array (**C**).

For dividing the sample, a microwell array chip consisting of 36 individual panels was designed (Fig. 1). Each panel consisted of a square array of 30 by 30 circular microwells (20 µm diameter) with a depth of 25 µm. Thus, the volume of an individual microwell is 1.257 × 10^−5^ µL and the total volume of all microwells 0.18 µL. Before conducting counting experiments, the well filling was tested using a fluorescein solution (Supplemental Figure S1). In rare cases, wells were not filled completely and air bubbles entrapped in the microwells. In other cases, repeated uses of the silicon molds lead to detachment of some of the individual microwell molds from the wafer. Furthermore, it could sometimes be observed that objects prevented complete sealing of the microwells from each other. However, filling analysis showed a high filling efficiency (>99% of wells filled) for two out of three devices, while the third device still had a filling efficiency of above 90% (Supplemental Figure S2). Looking at the filling of efficiency of the individual panels, a high filling efficiency was observed for all panels. For the third device, a PDMS particle prevented complete sealing of the microwell array to the glass slide in the top right corner. Through this, the solution from some of the wells in panel 5, 6, 11, and 12 was withdrawn when removing excess solution after filling. To remove variations from well filling, devices with entrapped air bubbles, missing microwells or other defects decreasing the filling efficiency below 99% were removed from further analysis.

**Figure 2:**
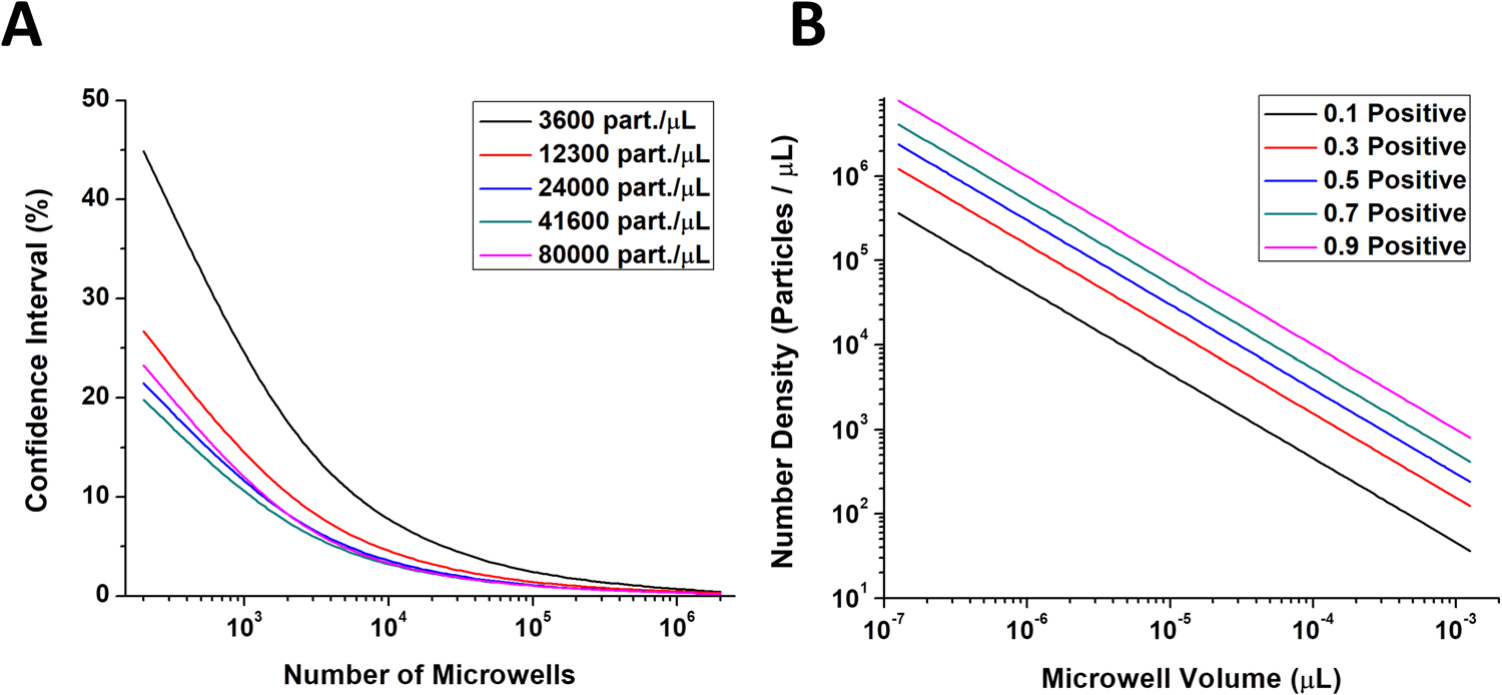
Analysis of microwell number and microwell volume on the counting performance of the device. Graph of the confidence interval as a percentage of the mean value against the number of wells for different number densities of particles (**A**). For this analysis, circular microwells with a diameter of 20 µm and a height of 25 µm were used. Graph of simulated number densities against the microwell volume for different fractions of occupied microwells (**B**).

A Poisson model was used to simulate the influence of microwell number and volume on the counting performance of the device (Fig. 2). Under the assumption of a Poisson distribution of particles into the wells, the number of microwells has no influence on the dynamic range of the device. However, the number of microwells is an important factor to affect the confidence interval of the measurement (Fig. 2A). The higher the number of microwells is analyzed, the smaller confidence interval is obtained. In a second simulation, the influence of microwell volume was analyzed (Fig. 2B). The microwell volume is an important factor influencing the range of number densities that can be measured by the device. While small microwells allow for the determination of high number densities in excess of 10^6^ particles/µL, wells with nanoliter volumes would allow counting of ten or less particles per microliter. However, in practice, there is a trade-off between these two factors. Increasing the well volume leads to a decrease in the number of wells per area and thus a larger confidence interval. Microfabrication, well filling, and imaging further limit the range of accessible well numbers and volumes. For the experiments conducted here, wells with a diameter of 20 µm were chosen with a height of 25 µm. This allows us to place a large number of wells (14,400) onto one device, while maintaining a geometry that can be reproduced with PDMS and SU-8.

### Algorithm for well recognition and fitting of well array

**Figure 3:**
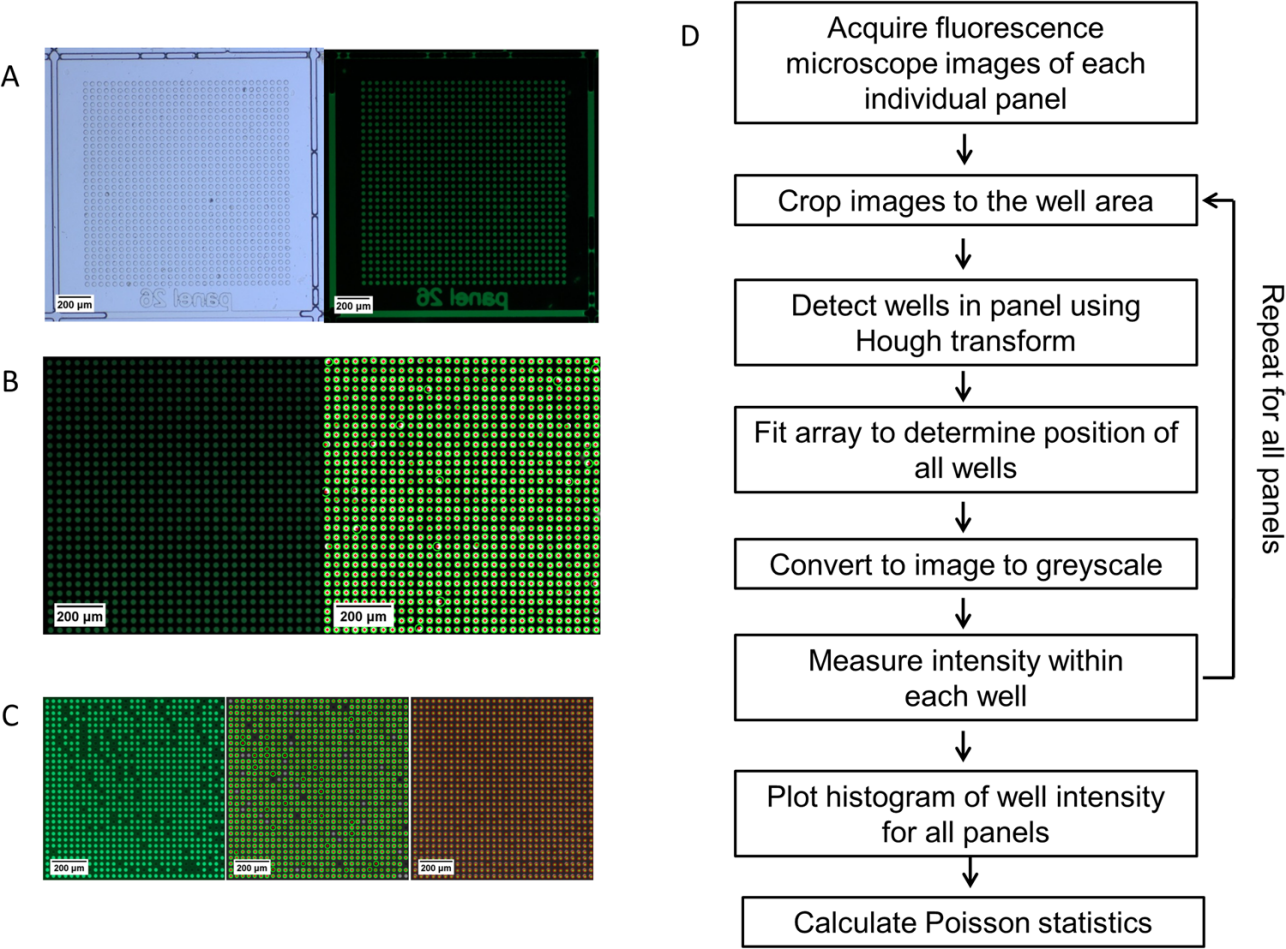
Illustration of well recognition and data analysis. Microscopy images of an individual panel filled with fluorescent dye in bright filed and fluorescent mode (**A**). Cropped well array of an individual panel filled with fluorescent dye (left) and wells identified by the Hough transform (right, **B**). Partially filled well array of a panel (left), wells identified by Hough transform (middle), and fitted well array (right, **C**). Flow chart of the algorithm used for the custom written python code for image analysis (**D**).

In a next step, the image recognition algorithm for the identification of wells and fitting of the microwell array was tested using microdevices filled with fluorescein solution (Fig. 3). Bright field and fluorescent microscopy images were taken of each individual panel of the microdevice (Fig. 3A) and the images then processed according to the proposed algorithm (Fig. 3D). Once the images were cropped to the area of the microwell array, the individual wells were identified using a Hough transform (Fig. 3B). When suitable parameters for the Hough transform were used, all wells could be reliably identified by the algorithm. Next, a purposefully incomplete filled microwell array was used to test the fitting of the microwell array (Fig. 3C). Using the position information of the wells found by the Hough transform, the complete array could be fitted. In addition to the test carried out with the fluorescent images, the same test repeated using the bright field images, yielding the same results (Supplemental Figure S3). A Hough transform was chosen due to its previously demonstrated robustness to random noise in the image^29^. However, noise originating from structured boundaries can lead to false recognition events. This can be reduced by setting boundaries to the Hough transform, so it only recognizes circular objects with radii within 1 µm of the radius of a microwell.

### Counting of microparticles

**Figure 4:**
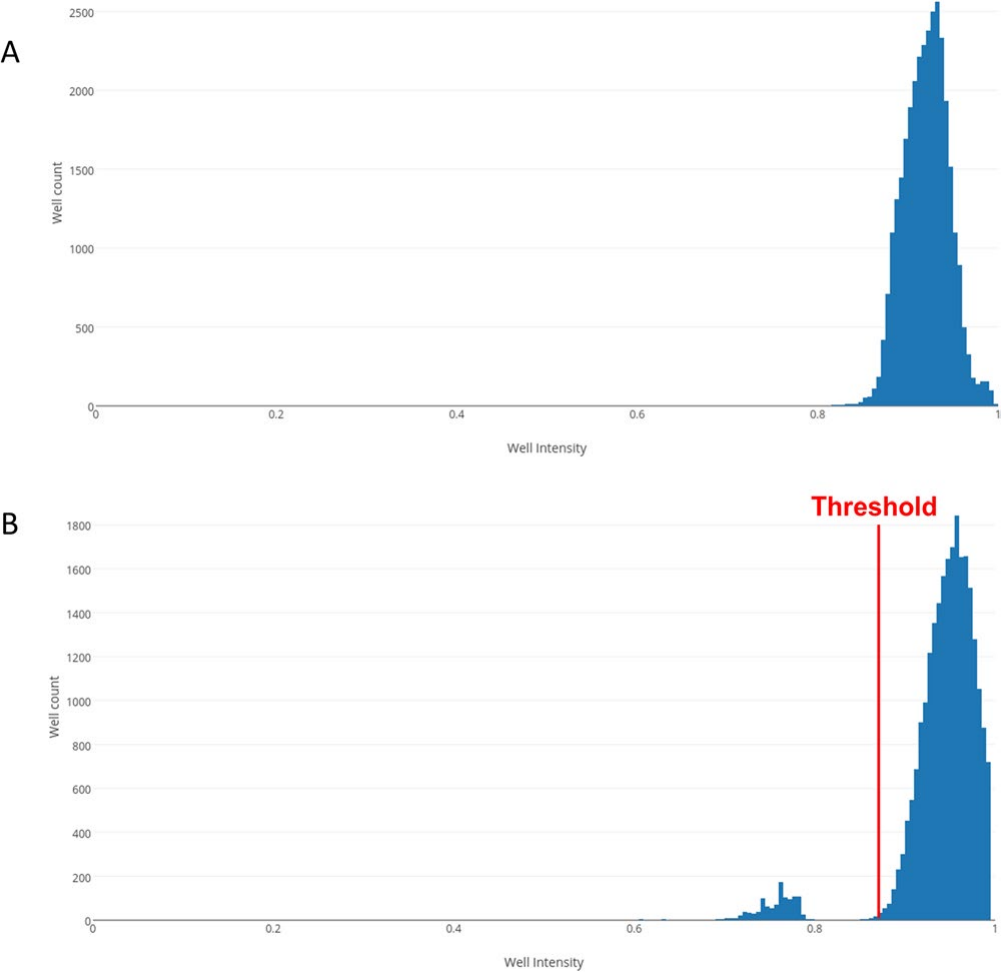
Histograms of well intensity for a control experiment with water (**A**) and a solution containing microparticles with a diameter of 10 µm (**B**).

**Figure 5:**
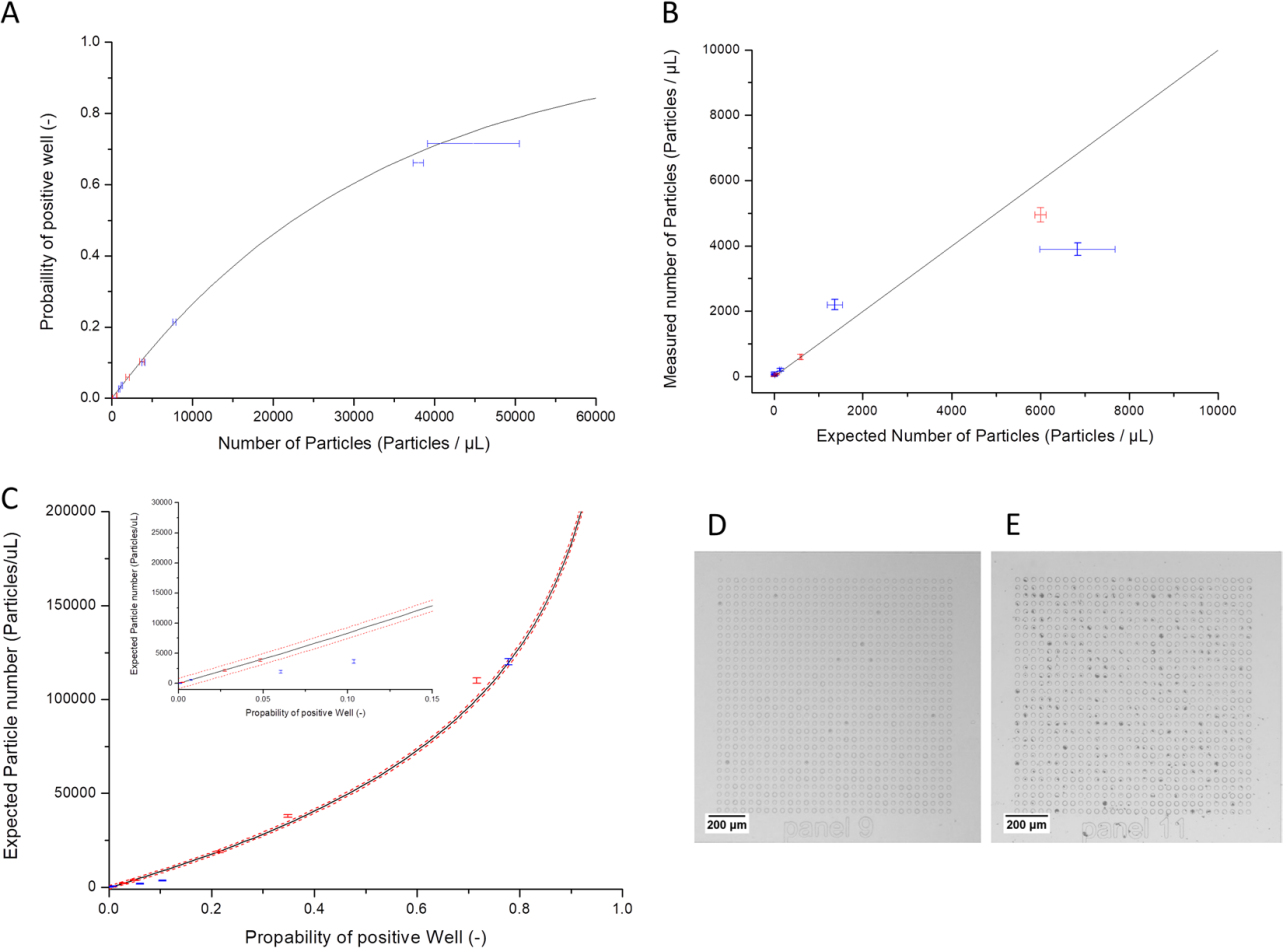
Graphs of number density of particles against the probability of finding an occupied well (**A**), expected number of particles against the measured number of particles (**B**), and the probability of an occupied well against the number of particles (**C**), with inlay showing the data at low probabilities. In all graphs, blue markers denote experiments with magnetic particles of diameter 5 µm, red markers are particles of diameter 10 µm, black lines are the values as expected from theory and the red lines in Fig. 4C is the theoretical standard deviation. The photographs show an image of a single panel when loaded with particles of diameter 10 µm (**D**) and magnetic particles of diameter 5 µm (**E**).

To count microparticles or cells using Poisson statistics, the ratio of microwells occupied by at least one particle or cell has to be determined. For this the microwells have to be classified into two groups. First, counting experiments were conducted with two different types of microparticles, fluorescent labeled polystyrene microparticle with a diameter of 10.2 µm and silica coated magnetic microparticles with a diameter of 1–5 µm. As transmission is blocked by the microparticles, microwells occupied by one or more particles displayed a lower light transmission compared to unoccupied wells. A histogram of the average pixel intensity of the wells fitted by the algorithm was made (Fig. 4). Occupied wells show as a second distinctive peak to the left of the peak of the unoccupied wells (Fig. 4B). A threshold was defined and the number of occupied and unoccupied determined from the histogram. Finally, the number of microparticles per microliter can be determined using Poisson statistics (Equations in Supplemental Materials). The particle counts obtained experimentally were compared to the theoretically obtainable values in first experiments (Fig. 5). For low number densities of the polystyrene and magnetic microparticles, the measured particle number coincided with the expected values (Fig. 5B). However, at large number densities (>50,000 particles/µL) the microdevice has a tendency to underestimate the particle number density. This is due to the microparticles forming aggregates and thus the occupation of the wells deviates from Poisson distribution. Due to the attraction of the magnetic microparticles, the effect is more prominent with these particles. In Fig. 5A it is shown that the probability of identifying a positive well is lower than expected from theory for high concentrations of magnetic microparticles, which directly results from the formation of aggregates. Furthermore, experimental variations, which were not considered in the theory, lead to further small deviations from the theoretically achievable accuracy (Fig. 5C). Here, microparticles of two different diameters were chosen for experiments. Particles of larger sizes could also be measured by the device, as long as their diameter is smaller than the microwell diameter. Furthermore, microwells cannot be occupied by two or more particles, which have a diameter that is only marginally smaller than the microwell diameter. Thus, at high number densities, these particles would not follow a Poisson distribution any more, increasing the error on these measurements. Particles with diameters smaller than 1 µm would not alter the transmission of the microwells enough to have a clear differentiation between occupied and unoccupied microwells. These small particles could be counted by decreasing the microwell diameter and using a higher magnification microscope, or by using fluorescent microscopy with strongly fluorescent nanoparticles.

**Figure 6:**
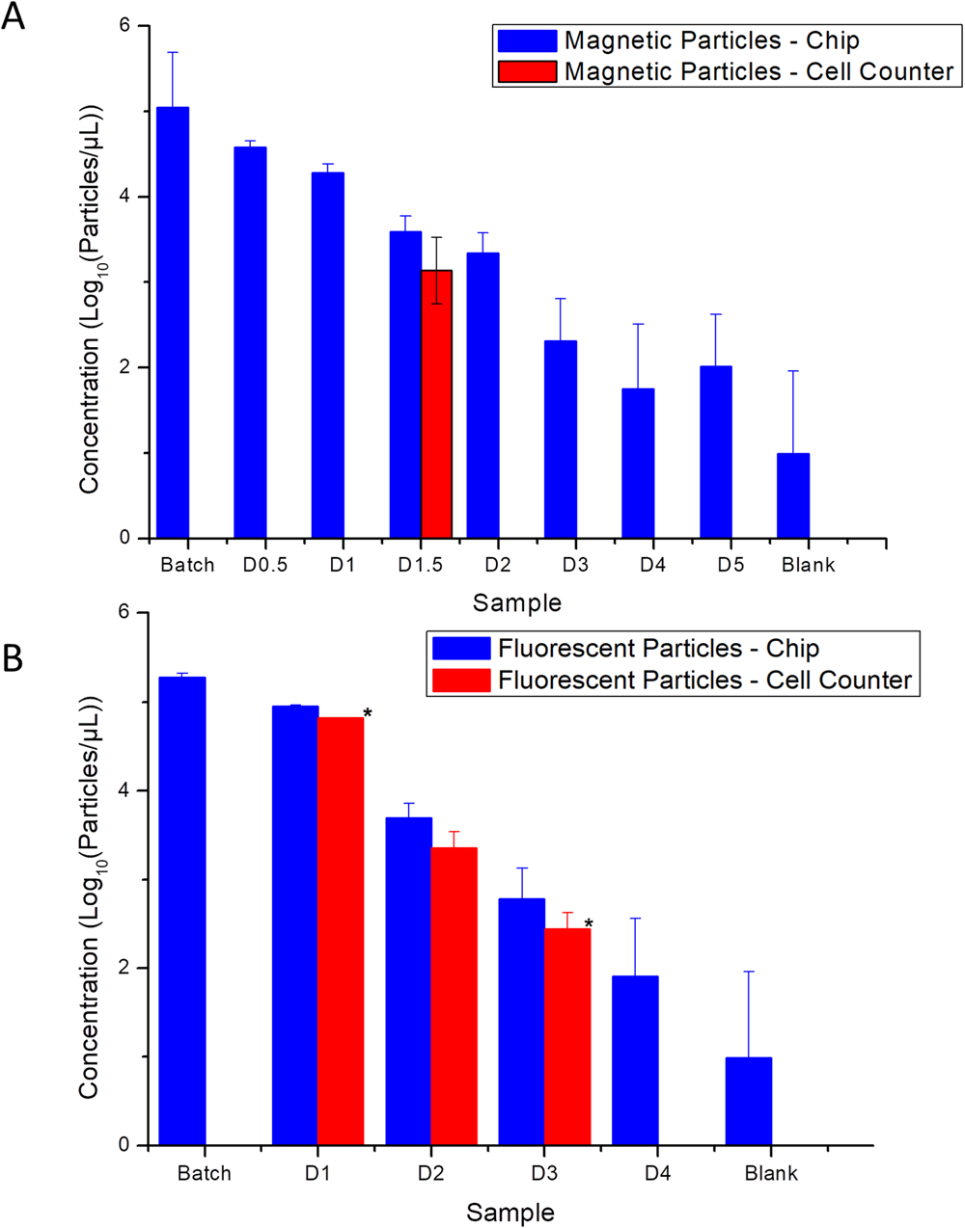
Determining of number densities by our method (blue) and a commercial cell counter (red) for comparison. Shown are different concentrations of magnetic particles with a diameter of 5 µm (**A**) and particles of diameter with a diameter of 10 µm (**B**). Batch samples were diluted by factors of 2 (D0.5), 10 (D1), 20 (D1.5),100 (D2), 1.000 (D3), 10.000 (D4), and 100.000 (D5). An asterisk (*) denotes measurements by the commercial cell counter that are indicated as outside the reliable measurement range by the device.

In an additional experiment, the counting performance of our PDMS microwell device was compared to a commercial device, using both particle types (Fig. 6). While the commercial device is only able to determine number densities across two orders of magnitude, our Poisson counting approach was able to measure number densities over three orders of magnitude (10^3^~10^5^ particles/µL). When approaching the limits of the detection range of the commercial device, the commercial device provides a lower or upper limit for the measurement. In addition, measurements with the commercial machine were severely limited by the formation of aggregates, as can be seen from Fig. 6A. The tendency of the magnetic particles to form aggregates decreased the accessible measurement range of the commercial device to determine particle numbers compared to the fluorescent particles. Our Poisson approach, in contrast, was not influenced by the aggregate formation and had the same measurement range for both particle types.

### Counting of Cells

**Figure 7:**
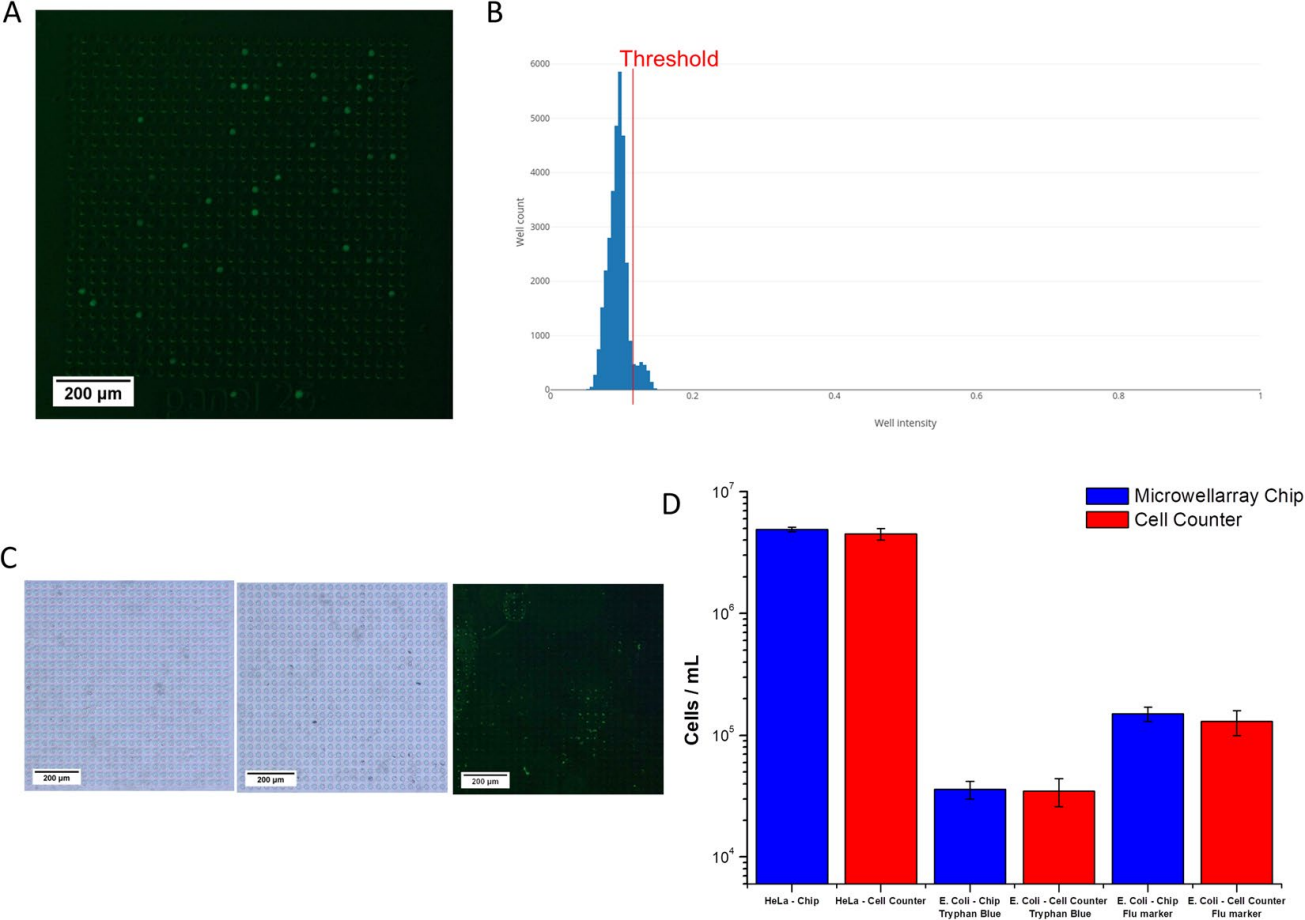
Determining of number densities of cells. Fluorescent microscopy image of a single panel filled with GFP labeled HeLa cells (**A**). Histogram of well intensity for all 36 panels, determining the number of GFP labeled HeLa cells (**B**). Microscopy images of a single panel containing unlabeled (left), Trypan Blue labeled (middle), and fluorescent labeled *E*. *Coli* (right, **C**). Bar diagram of determined number densities of *HeLa* cells and *E. Coli* stained using Trypan blue and fluorescent staining (blue), and comparison to commercial cell counter (red, **D**).

In addition to the counting of microparticles, counting of cells using Poisson statistics and the microwell PDMS array was also demonstrated with *E*. *Coli* and *HeLa* cultures (Fig. 7). Due to the high light transmittance of cells, a clear classification of unoccupied and unoccupied wells was more difficult. To allow for easier classification cells have to be stained first using a dye, such as Trypan blue (Fig. 7C), in which case analysis can be conducted as previously done with microparticles. Alternatively, the cells can be marked fluorescently, as done with the GFP expressing HeLa cells (Fig. 7A), or by using a fluorescent dye, such as BacLight Green Bacterial Stain (Fig. 7C). As the classification of wells into the two groups is dependent on the staining of the cells, the accuracy for the cell counting is also dependent on an efficient cell staining protocol. When a fluorescent dye is used, the emission of the fluorophore shows occupied wells as bright spots on a dark background in fluorescent microscopy. Therefore, creating a histogram of well fluorescent intensity, occupied wells indicate as a second peak with higher intensity compared to unoccupied wells (Fig. 7B). After classification of wells into the two groups, analysis could be conducted as before using Poisson statistics. The number density of three different culture samples was analyzed and compared to measurements from a commercial cell counter (Fig. 7D); one sample containing GFP expressing *HeLa* cells, one *E*. *Coli* sample stained with Trypan blue, and a second *E*. *Coli* sample stained with BacLight Green Bacterial Stain. The number densities measured by our method and the commercial cell counter were identical for all samples. However, the confidence interval for the measurement using our Poisson method were around 60% smaller compared to the values from the commercial device. We reckon that this difference originates from the method of image analysis. While the commercial cell counter searches the images for individual cells, our method searches for the microwells. Hence, with an irregular-shaped sample the image recognition algorithm of the commercial device is going to show a higher variance, while our algorithm searches for the microwells, which are of regular shape and size, as defined by fabrication. Surprisingly, 10^6^ cells/µL could be accurately counted with our method, while only 10^5^ particles/µL could be counted using the same method. This observation can be explained by the lower tendency of the cells to form aggregates, compared to the microparticles. Therefore, the assumption of a Poisson distribution into the wells is valid at higher number densities compared to microparticles.

## Conclusions

We demonstrated a new method for determining the number density of microparticles or cells using Poisson statistics and microwell arrays. The method is able to accurately determine the number density over a range spanning several orders of magnitude. As the method is working with an image recognition algorithm recognizing the microwells, which are of specific size and shape defined by fabrication, the method is more robust regarding irregular-shaped samples and aggregates compared to other image recognition based methods. The method can be conducted with only basic laboratory equipment and is fast, simple, and inexpensive to conduct. These advantages make our method particularly interesting for resource limited applications or point-of-care applications.

## Electronic supplementary material

Supplemental Information
