# Supplementary material for: Poisson statistics-mediated particle/cell counting in microwell arrays
Source: Sci Rep. 2018 Feb 5;8:2438. doi: 10.1038/s41598-018-20913-0 (PMC5799205; doi:10.1038/s41598-018-20913-0)
Supplement: Supplementary file 1 — Supplemental Information [file 41598_2018_20913_MOESM1_ESM.pdf]

# Poisson statistics-mediated particle/cell counting in microwell arrays

Christian D. Ahrberg, Jong Min Lee, Bong Geun Chung*

Department of Mechanical Engineering, Sogang University, Seoul, Republic of Korea

## Supplemental Figure and Table

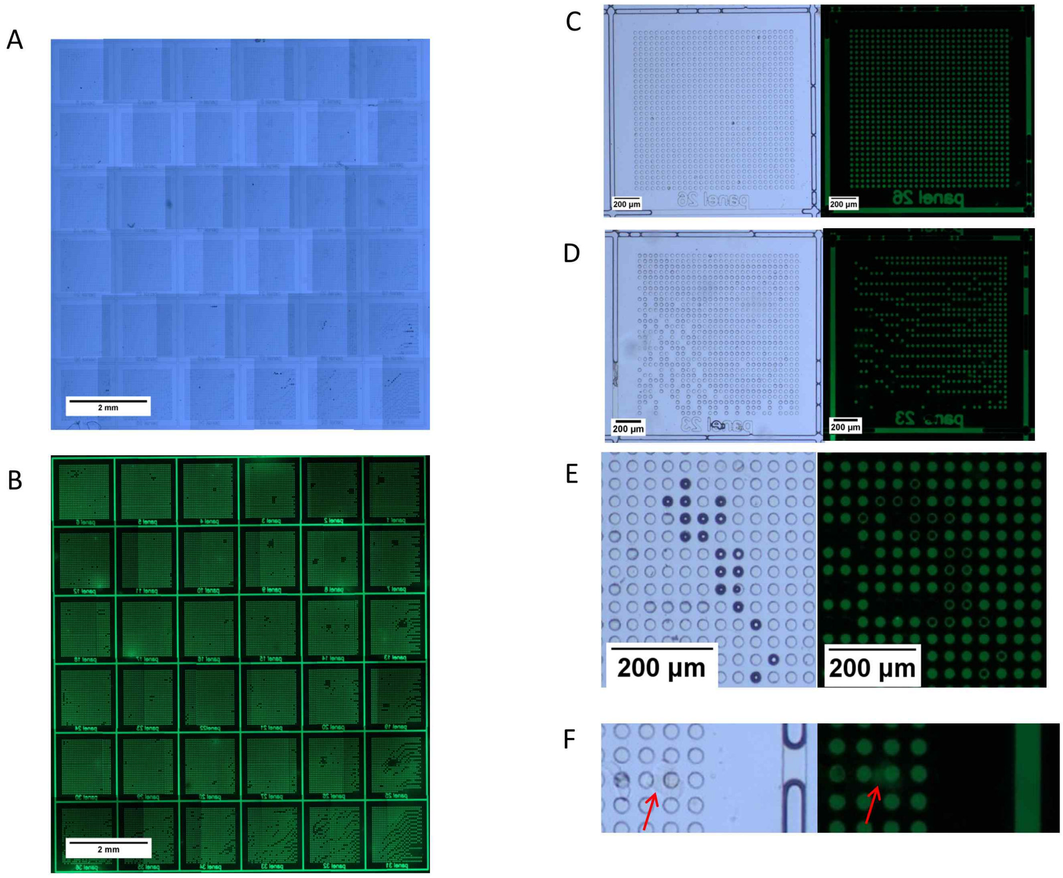


Supplemental Figure S1. Microscopy images of entire device filled by fluorescent dye in bright field settings (A) and fluorescent mode (B). Example image of panel completely filled with fluorescent dye in bright field and fluorescent mode (C). Example of filling errors that can arise as observed by bright field and fluorescent microscopy: Defective PDMS mold missing wells (D), air bubbles trapped in the wells during the filling process (E), and cross-talk between wells caused by entrapped dirt fragments (F).

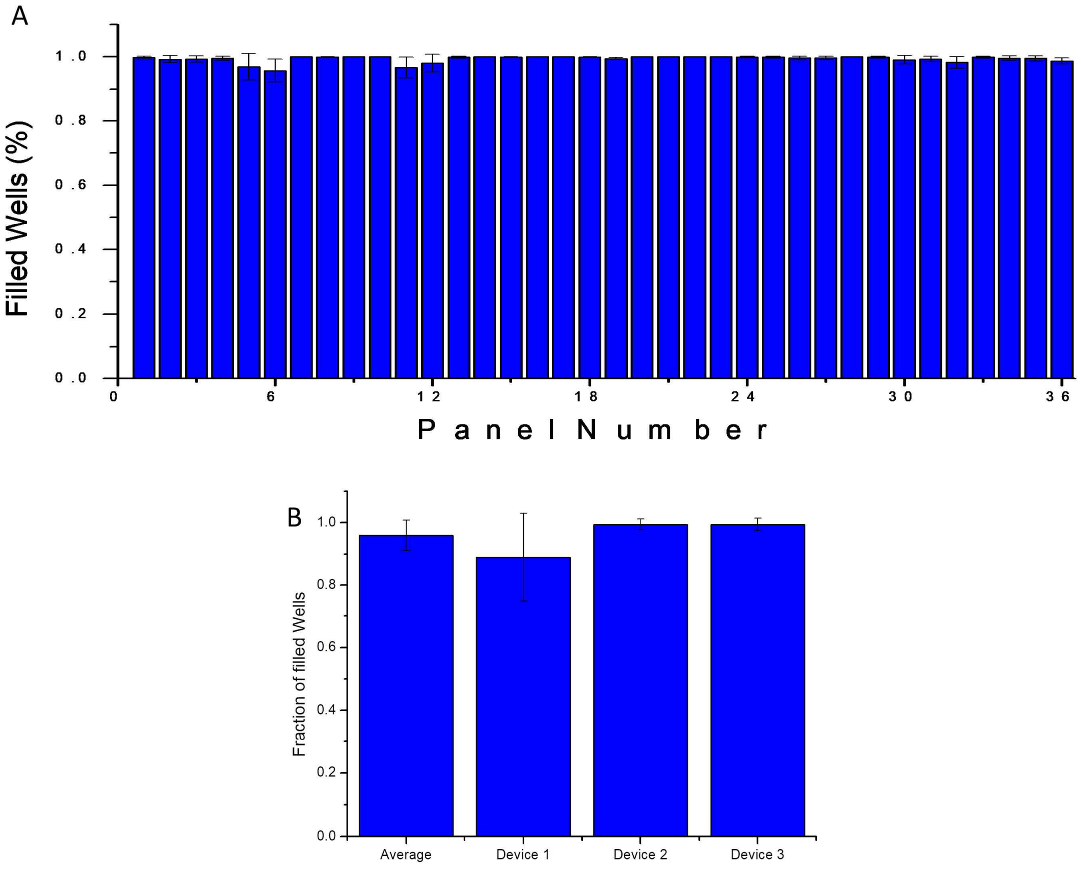


Supplemental Figure S2. Average fraction of filled wells for individual panels as the average of three independent devices (A) and the average of wells filled for each of the three devices as an average of all panels (B).

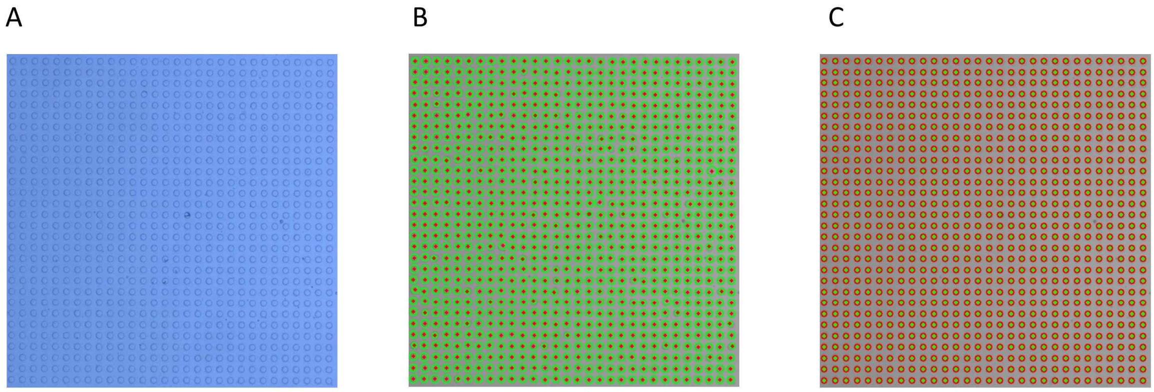


Supplemental Figure S3. Illustration of well recognition and data analysis using bright field images. Microscopy image of one panel of the microwell array (A), microwells identified by Hough-Transform algorithm (B), and microwell array fitted by algorithm (C).

| **Step Name** | **Action** |
|---|---|
| Pretreatment | Clean wafers at 200°C for 5 min |
| Spin Coating | 1. Place 5mL of SU-8 50<br>2. Ramp to 500 rpm with slope of 100 rpm/sec<br>3. Ramp to 1,000 rpm with slope of 300 rpm/sec<br>4. Hold for 60 sec |
| Soft Bake | 1. Pre-bake at 65°C for 10 min<br>2. Soft bake at 95°C for 40 min |
| Exposure | Expose to UV using photomask for 15 sec |
| Post Bake | 1. 1 min at 65°C<br>2. 10 min at 95°C |
| Develop | Develop with SU-8 Developer for 10 min<br>(ethyl lactate/diacetone alcohol) |
| Rinse and Dry | Rinse with i-Propanol, dry with nitrogen gas |

Supplemental Table S1. Fabrication steps for silicon masters.

**Supplemental Materials**

**Poisson Distribution**

Particles and cells are distributed into wells according to a Poisson distribution:

$$P_\lambda(k) = \frac{\lambda^k}{k!} e^{-\lambda} \qquad (1)$$

Where P is the probability of having k particles or less inside of a well. λ is the average number of particles or cells per well. Therefore, the probability of having an empty well can be expressed as follows:

$$P_\lambda(k = 0) = \frac{\lambda^0}{0!} e^{-\lambda} = e^{-\lambda} \qquad (2)$$

From a measurement the probability of having an empty well can be estimated as follows:

$$\hat{P}(k = 0) = 1 - \frac{N_P}{N_W} \qquad (3)$$

where $N_P$ is the counted number of positive wells, containing a particle or cell, and $N_W$ is the number of total wells. The confidence intervall for the probability can be estimated using a Z-distribution in the following way:

$$\sigma_{\hat{P}}^2 = 1.96\sqrt{\frac{\hat{P}}{N_W}} \qquad (4)$$

Where 1.96 is the Z-score for a 95% confidence interval. The number of particles or cells can now be estimated by subtitling $\hat{P}$ into eqn. 2.

**Python code for data analysis:**

The python code opens a series of jpg images, with numerical ascending image (e.g. 1.jpg, 2.jpg, 3.jpg,…) names saved in the same folder as the Python file. The code then automatically detects wells in the images and outputs the histogram of well intensity. After a threshold value is manually entered by the user, the program calculates the number densities, according to the histogram. Parameters for well identification, the number of images, well size, and array size can be entered at the beginning of the Python code. In addition to the histogram, the code also saves additional files in the folder it is executed. A list of these files, can be found in the beginning comments of the code.

The Python code can be found in a public git repository under the following link:
https://github.com/cahrberg/Counting-Cells-Particles/blob/master/Grid_Intensity_circles_v3.py
